# LXW7 Peptide Modification of Acellular Liver Scaffolds Improves Endothelialization and Hemocompatibility in Bioengineered Liver

**DOI:** 10.3390/jfb17030122

**Published:** 2026-03-03

**Authors:** Usha Yadav, Chandra J. Yadav, Sadia Afrin, Jun-Yeong Lee, Jihad Kamel, Kyung-Mee Park

**Affiliations:** College of Veterinary Medicine, Chungbuk National University, Cheongju 28644, Republic of Korea; ushacj.23@chungbuk.ac.kr (U.Y.); chandrajit@chungbuk.ac.kr (C.J.Y.); sadiaafrin1406@gmail.com (S.A.);

**Keywords:** bioengineered liver, LXW7, endothelialization, hemocompatibility, fibrosis

## Abstract

End-stage liver disease caused by advanced fibrosis and cirrhosis remains a major global burden, yet its treatment is limited by donor organ shortages. Bioengineered liver scaffolds offer a promising alternative, but their efficacy is often limited by thrombosis, insufficient vascularization, and poor graft integration due to inadequate endothelialization. To overcome these challenges, we employed LXW7 αvβ3 integrin targeting peptide with high endothelial cell specificity and low platelet affinity to enhance re-endothelialization and hemocompatibility of decellularized liver scaffold (DLS) and thereby improve hepatic integration and function. LXW7 was covalently conjugated to the decellularized rat liver scaffold via EDC/NHS-mediated carbodiimide coupling and subsequently reseeded with human umbilical vein endothelial cells (HUVECs) and cultured in a perfusion bioreactor to promote endothelialization. LXW7 immobilization significantly improved HUVECs attachment and proliferation, achieving approximately 81% vascular coverage, while sustaining the endothelial function. Ex vivo blood perfusion showed minimal thrombus formation and markedly reduced platelet adhesion, demonstrating enhanced hemocompatibility. Following confirmation of endothelialization, scaffolds were recellularized with hepatocellular carcinoma (HepG2) cells and HUVECs. LXW7 modified scaffolds promote organized hepatocyte distribution, sustained albumin expression, and increased urea secretion. In vivo implantation of LXW7-DLS into the omentum of mice promoted robust host endothelial recruitment and enhanced neovascularization, highlighting the scaffold’s excellent biocompatibility and good integration with surrounding tissues. Moreover, in vivo implantation of LXW7 recellularized scaffolds into a thioacetamide-induced fibrotic mouse liver resulted in reduced collagen deposition and lowered serum ALT/AST levels, demonstrating hepatic regeneration and extracellular matrix remodeling. Overall, our results showed that LXW7-modified DLS promotes stable endothelialization, improves hemocompatibility, and enhances hepatic function, underscoring its translational potential for the development of vascularized transplantable liver grafts.

## 1. Introduction

Liver transplantation is a life-saving intervention and remains the gold standard curative therapy for patients with end-stage liver disease, including decompensated fibrosis and cirrhosis. Despite its clinical success, transplantation is severely limited by the global shortage of donor organs, with less than 10% of the demand being met, leading to prolonged waiting lists and high mortality among patients awaiting treatment [1]. Tissue engineering may offer alternative solutions to cover the donor organ gap by creating artificial tissues.

Tissue engineering, a leading field within regenerative medicine, seeks to combine scaffolds, cells, and bioactive molecules to generate functional tissues capable of restoring, maintaining, or enhancing damaged organs [2]. Within this context, whole-organ bioengineering has emerged as a particularly promising strategy, aiming to develop fully functional, clinically relevant, and implantable organs. This approach typically employs native organs from xenogeneic sources, in which cellular components are removed through decellularization while preserving the essential structural, mechanical, and biochemical characteristics of the original tissue. Such preserved extracellular matrices (ECM) provide a biologically and mechanically supportive framework for subsequent recellularization, ultimately enabling the reconstruction of complex organ architecture and function [3,4,5].

Although considerable progress has been made in liver tissue engineering, bioengineered livers are not yet ready for clinical transplantation due to remaining biological and translational challenges. Among them, re-establishing vascular patency represents a major challenge as thrombosis and perfusion failure frequently compromise the graft viability [6,7]. The successful endothelialization of the decellularized liver scaffold (DLS) is critical for re-establishing vascular function, providing an antithrombogenic surface, regulating permeability, and supporting physiological blood flow following implantation [8,9]. Nevertheless, achieving complete and stable endothelial coverage throughout the complex vascular network of the liver has proven difficult, even with repeated seeding and perfusion strategies. In this context, robust endothelial cells (ECs) attachment to the scaffold surface is a critical prerequisite, as it determines subsequent cell proliferation, migration, and the establishment of a stable, functional endothelial lining.

Over the years, numerous functional molecules and capture ligands have been investigated for ECs attachment, like ECs-related growth factors, peptides, antibodies, aptamers, oligosaccharides, proteins, and magnetic molecules [10,11,12,13,14,15]. However, many of these are structurally unstable, lack specificity for ECs, or provide only passive capture without actively promoting endothelial functions. Among them, the ECM-derived integrin αvβ3-binding RGD peptide has been widely applied to enhance ECs’ adhesion. Despite its ability to enhance cell adhesion, RGD lacks specificity for ECs [16,17] and can also recruit platelets [18,19,20,21] and inflammatory cells [21,22] increasing the risk of thrombosis and inflammation, thereby hindering clinical translation. These drawbacks highlight the critical need for alternative ligands with higher ECs specificity and functional activity, while preventing platelet and inflammatory cell accumulation.

LXW7, an integrin αvβ3 ligand, possessed strong, stable, and specific ECs capturing function, supporting targeted adhesion, proliferation, and angiogenesis [17,21]. LXW7 possessed the highest binding specificity to endothelial progenitor cells (EPCs)/ECs. ECs bound to an LXW7-treated culture surface exhibited enhanced biological functions such as proliferation, due to increased phosphorylation of VEGF receptor 2 (VEGF-R2) and activation of mitogen-activated protein kinase (MAPK) ERK1/2 [17,23]. Unlike the conventional αvβ3-binding RGD peptide, which binds substantially to platelets and increases thrombosis risk. LXW7 demonstrates markedly weaker platelet interaction, significantly higher affinity for EPCs and ECs, and no detectable binding to inflammatory monocytes [17]. Beyond its superior specificity and functionality, LXW7 also possesses enhanced structural and proteolytic stability, owing to its cyclic structure and incorporation of four unnatural D-amino acids, making it particularly suitable for in vivo applications [21]. These unique characteristics render LXW7 a highly promising ligand to facilitate EPCs/ECs attachment, function, and vascular integration in engineered tissue constructs. The balance of cell–matrix interactions, mediated by adhesion molecule signaling, is fundamental for achieving stable endothelialization. Previous approaches, including anti-endothelial antibodies [24], heparin-based modification [25,26] and REDV peptides [27] have been employed to enhance vascularization in DLS. Although these strategies improved ECs’ adhesion, proliferation, and thromboresistance to varying degrees, achieving complete and durable endothelial coverage across the entire vascular network remains a major challenge.

In this study, LXW7 was covalently conjugated to the DLS via EDC/NHS-mediated carbodiimide coupling, resulting in amide bond formation to enable selective binding to αvβ3-integrin on human umbilical vein endothelial cells (HUVECs), thereby promoting EC attachment while minimizing platelet adhesion. This dual functionality supports efficient re-endothelialization and simultaneously improves hemocompatibility. By leveraging LXW7 unique ECs specificity, functional stability, and anti-thrombogenic profile, this strategy addresses a critical limitation in liver tissue engineering by enabling the establishment of functional vascularization. Consequently, it advances the development of clinically translatable bioengineered liver constructs.

## 2. Materials and Methods

### 2.1. Cell Culture

HUVECs (C-12200) were obtained from Promo Cell (Heidelberg, Germany) and cultured according to the manufacturer’s instructions. Cells were thawed and maintained in Endothelial Cell Growth Medium-2 (EGM-2, C-22011, Promo Cell) supplemented with the Endothelial Cell Growth Medium-2 Supplement Mix (C-39216, Promo Cell), and cells from passages 4–5 were used for experiments. Hepatocellular carcinoma (HepG2) cells were purchased from ATCC (Manassas, VA, USA) and grown in high-glucose Dulbecco’s modified Eagle medium (DMEM; Welgene, Gyeongsan, Republic of Korea) supplemented with 10% fetal bovine serum (FBS, Hyclone, Logan, UT, USA) and 1% antibiotic-antimycotic (ABAM, Welgene, Gyeongsan, Republic of Korea). Cells were sub-cultured and maintained at 37 °C in a humidified incubator with 5% CO_2,_ and cell passages 17–19 were used for experiments.

### 2.2. In Vitro Cell Attachment Assay

The LXW7 (sequence: CGRGDDDVDVCK(FITC)-NH_2_) was custom synthesized by Peptron Co., Ltd. (Daejeon, Republic of Korea). To evaluate HUVECs attachment, FITC-labeled LXW7 at different concentrations of 1, 2, and 5 μM was either directly applied onto a gelatin-coated plate and incubated for 2 h (adsorption method) or covalently conjugated to gelatin by EDC/NHS coupling chemistry (immobilization method). For immobilization, 1% gelatin-coated wells were treated with a solution containing 0.05 M 1-Ethyl-3-(3-dimethylaminopropyl) carbodiimide hydrochloride (EDC; Sigma-Aldrich, St. Louis, MO, USA) and 0.06 M N-hydroxy succinimide (NHS; Sigma-Aldrich, St. Louis, MO, USA) in 0.05 M MES buffer (pH 5.6) for 30 min at room temperature. Subsequently, 1, 2, or 5 μM of LXW7 peptide dissolved in phosphate-buffered saline (PBS) was applied to the EDC/NHS-coupled gelatin plate and incubated for 2 h at room temperature. Unreacted peptide was removed by washing with PBS. HUVECs (2 × 10^5^) were seeded onto 24-well plates coated with either LXW7 modified gelatin or unmodified gelatin (negative control). After 2 h, the culture medium was removed to eliminate non-adherent cells. Wells were washed with PBS, fixed in 4% paraformaldehyde (PFA) for 10 min, and stained with 0.1% crystal violet (Sigma-Aldrich) and CD31/DAPI (4′,6-diamidino-2-phenylindole) for nuclear visualization. Cell attachment and spreading were examined under an Optinity KCS3-50SS microscope (Korea Labtech, Seongnam, Republic of Korea). Quantitative analysis of DAPI-positive adherent cells per field at 20× magnification was performed using the ImageJ 1.54d. To ensure consistency and reduce bias, all images were processed using the same threshold settings and analysis parameters. HUVECs’ proliferation and viability on different LXW7 concentrations were assessed on days 1, 2, and 3 using a cell counting kit-8 (CCK-8) (Sigma-Aldrich, MO, USA). To further evaluate the capacity of LXW7 immobilized DLS to support HUVECs attachment, 10 mm slices of DLS were prepared using a biopsy punch (Acuderm^®^ Inc., FL, USA). DLS samples were conjugated with FITC-labeled LXW7 via EDC/NHS-mediated carbodiimide coupling, resulting in amide bond formation, while DLS without any treatment served as negative controls. After the PBS washing, 500 μL of a suspension containing 5 × 10^4^ HUVECs was seeded onto the scaffold surface. Following 2 h of incubation, adherent cells were evaluated by immunofluorescence (IF) staining. DLS slices were washed with PBS, fixed in 4% PFA for 10 min, and stained with CD31/DAPI. Adherent cells were quantified by counting DAPI-positive cells per field at 20× magnification using the ImageJ software. CCK-8 assay was performed for cell viability and proliferation analysis.

### 2.3. Rat Liver Procurement and Decellularization

Decellularization of rat livers was performed using our previously established protocol with minor modifications [28,29]. Prior to the experiment, all animals were given a seven-day acclimation period in the laboratory and housed at 25 °C under a controlled 12 h light-dark cycle. Livers were harvested from 10-week-old female Sprague Dawley rats (250–300 g). Rats were deeply anesthetized by intraperitoneal (IP) injection of Alfaxan (30 mg/kg) and Domitor (0.5 mg/kg), after which a U-shaped incision was made to expose the liver. The PV was cannulated with a 24-G catheter and the bile duct with a 26-G catheter, and the liver was perfused with 60 mL of heparinized PBS (10 IU/mL) to ensure complete removal of blood. The abdominal aorta and superior vena cava were transected prior to liver separation, resulting in euthanasia. Following dissection, the livers were perfused with heparinized PBS for 60 min at 2 mL/min using a peristaltic pump (JWTE 600, JenieWell, Seoul, Republic of Korea). Decellularization was performed with perfusing 1% Triton X-100 and 0.1% ammonium hydroxide (Samchun Chemical, Seoul, Republic of Korea) at 3 mL/min for 8 h. The scaffolds were subsequently washed with PBS for 12 h, sterilized with 0.1% peracetic acid, and stored in PBS supplemented with antibiotics at 4 °C until use. All animal experiments were approved by the Institutional Animal Care and Use Committee of Chungbuk National University (CBNUA-24-0091-01).

### 2.4. Characterization of Decellularized Liver Scaffold

Histological analysis was conducted on native and decellularized livers to evaluate cellular removal and ECM retention. Samples were fixed in 10% neutral-buffered formalin, processed for paraffin embedding, and sectioned at a thickness of 5 µm. The sections were subsequently deparaffinized and stained with hematoxylin and eosin (H and E) and with DAPI, to confirm the complete elimination of nuclear material. For ultrastructural evaluation, samples were fixed overnight in 2.5% glutaraldehyde, post-fixed in osmium tetroxide, dehydrated in graded ethanol, sputter-coated with gold palladium, and examined by scanning electron microscopy (SEM; Gemini 560, Oberkochen, Germany). Quantitative analysis of DNA content in native and decellularized rat livers (*n* = 5) was performed using the G-spin™ Total DNA Extraction Kit (Intron Biotechnology, Gyeonggi, Republic of Korea), following the manufacturer’s protocol. Preservation of key ECM components of DLS was evaluated by immunohistochemistry (IHC) for collagen type IV, laminin, and fibronectin on paraffin-embedded sections. Additionally, the vascular network of the DLS was assessed by perfusion with 0.1% crystal violet through PV to visualize vascular patency.

### 2.5. Immobilization and Characterization of LXW7 Modified DLS

LXW7 peptides were covalently conjugated to DLS using EDC/NHS-mediated carbodiimide coupling. Briefly, a 50 mL solution containing 0.05 M EDC and 0.06 M NHS in 0.05 M MES buffer (pH 5.6) was perfused through the portal vein (PV) and incubated for 30 min at room temperature. Subsequently, 50 mL of PBS containing 1 μM LXW7 peptide was perfused into the scaffold and incubated for 4 h at room temperature. Scaffolds were then washed with 30 mL of PBS to remove unbound peptides. The FITC-labeled LXW7 immobilized scaffolds were characterized by direct fluorescence microscopy (Korea LabTech, KI-3000, Gyeonggi, Republic of Korea), Fourier-transform infrared spectroscopy (FTIR; Agilent Cary620, Santa Clara, CA, USA), and Energy-dispersive X-ray spectroscopy (EDS; JEOL JXA-IHP200F, Peabody, MA, USA) to confirm peptide conjugation and scaffold modification. Antiplatelet properties of the LXW7 immobilized surface were assessed by the Platelet Attachment Assay (Supplementary Method S1).

### 2.6. Recellularization of DLS

#### 2.6.1. Re-Endothelialization of DLS with HUVECs

Re-endothelialization of DLS and LXW7 modified liver scaffolds (*n* = 3 scaffolds/group) was performed using HUVECs. Initially, both DLSs were perfused with EGM-2 medium for 45 min to stabilize the ECM. A total of 2 × 10^7^ cells were introduced via the PV in four consecutive infusions, each delivering 5 × 10^6^ cells/mL at 10 min intervals. Following seeding, the constructs were maintained under static conditions for 3 h to facilitate initial cell attachment. Thereafter, the scaffolds were transferred to a perfusion bioreactor and cultured for 7 days under standard conditions (37 °C, 5% CO_2_) with continuous medium flow at 2 mL/min. Culture medium was replaced daily, and perfusate was collected and stored at −80 °C for subsequent biochemical analyses. At the end of the 7-day culture period, the re-endothelialized scaffolds were collected for both histological examination and quantitative analyses. Quantitative analyses were performed to assess endothelial coverage by counting three different microscopic fields per slide from five different lobes, for cellular distribution, and the functional status of the reseeded constructs. CD31 immunostaining was performed to confirm endothelial identity and to verify vascular lining formation within the scaffold. Nitric oxide (NO) secretion was quantified using the NO Plus Detection Kit (Intron Biotechnology, Gyeonggi, Republic of Korea) in accordance with the manufacturer’s recommended protocol.

#### 2.6.2. Recellularization of DLS with HepG2 and HUVECs Cell

Both LXW7 modified and unmodified DLS (*n* = 3 scaffolds) were repopulated with hepatocytes, followed by HUVECs. HepG2 cells are used as a source for primary hepatocytes due to their ready availability, stable hepatic phenotype, and partial preservation of genotypic and phenotypic characteristics of normal liver cells. For hepatocyte seeding, a total of 6 × 10^7^ HepG2 cells were introduced into the parenchymal regions through both the PV and the bile duct. Cells were delivered in six sequential infusions at a 5 × 10^6^ cells/mL density. To achieve uniform distribution, half of the total cell (3 × 10^7^) was infused via the PV and the remaining (3 × 10^7^ cells) through the bile duct. After seeding, constructs were maintained under static conditions for 3 h for initial attachment and subsequently connected to a perfusion bioreactor. After 24 h of hepatocyte culture, endothelialization was performed by infusing 2 × 10^7^ HUVECs into the vasculature via the PV in four consecutive injections. The co-culture constructs were maintained under dynamic perfusion for 7 days. Culture medium was replaced daily, and perfusate samples were collected and stored at −80 °C for biochemical analyses. Hepatocellular function was quantified by measuring urea concentrations in the perfusate using a Urea Assay Kit (ab83362, Abcam, Cambridge, UK) according to the manufacturer’s protocol.

### 2.7. Resazurin Reduction Assay of Re-Endothelialized Scaffold

Cell viability and proliferative activity within the re-endothelialized liver constructs (*n* = 3) were assessed using a resazurin-based metabolic assay performed on alternate days throughout the 7-day culture period. A 440 μM resazurin stock solution (10×) of Resazurin sodium salt (R7017, Sigma-Aldrich) was prepared in PBS [30]. For each assay, a 44 μM working solution was prepared by diluting the stock solution 1:10 in EGM-2 medium. On days 1, 3, 5, and 7, each construct was perfused with 80 mL of the resazurin working solution at 2 mL/min for 1 h at 37 °C. Following perfusion, images of the resazurin-reduced scaffolds were taken to document color changes, after which the resazurin solution was replaced with fresh medium. The absorbance of the perfused solution was measured at 570 nm and 600 nm to quantify the metabolic conversion of resazurin. The percentage reduction, indicative of HUVECs’ metabolic activity and proliferation, was calculated.

### 2.8. Thrombogenic Evaluation of Re-Endothelialized Constructs

To evaluate the thrombotic response and efficiency of endothelialization, re-endothelialized liver scaffolds (*n* = 3) were retrieved from the perfusion bioreactor after 7 days of dynamic culture and subsequently perfused with porcine blood. Fresh porcine blood collected from a local slaughterhouse was anticoagulated with sodium citrate and diluted 1:1 with PBS for perfusion. The constructs were perfused with diluted blood for 1 h, after which they were thoroughly rinsed with PBS to remove non-adherent components. The macroscopic photographs of the scaffolds were captured to visualize the clot formation within the scaffold. Platelet deposition on the scaffold surface was assessed by IF staining using an anti–integrin αIIb antibody. Quantification of platelet adhesion was performed by measuring integrin αIIb fluorescence intensity from randomly selected microscopic fields using ImageJ software. Blood samples were collected from the perfusion at predefined time points (0, 15, 30, and 45 min) during ex vivo blood perfusion. Platelet counts were measured using an automated hematology analyzer following standard operating procedures. The platelet numbers at each time point were expressed as percentages to evaluate time-dependent changes in platelet count.

### 2.9. Omentum Implantation

An in vivo angiogenesis assay was performed using Balb/c nude male mice aged 6 weeks (Nara-Biotec, Seoul, Republic of Korea) to evaluate the angiogenic potential of DLS and LXW7-DLS scaffolds. Following intraperitoneal anesthesia with Alfaxan (30 mg/kg) and Domitor (0.5 mg/kg) and aseptic preparation, 10 mm scaffold disks from both groups were implanted into the omentum (*n* = 4 mice per group per time point) and maintained in vivo for 21 days. Following surgery, meloxicam (5 mg/kg) was administered subcutaneously once daily for three days to ensure postoperative pain relief. In addition, postoperative pain was monitored based on behavioral and clinical indicators, in accordance with institutional animal welfare guidelines. At the predetermined endpoint, mice were humanely euthanized using a lethal dose of anesthesia, and gross images of the implanted scaffold were captured to document the formation of neovascularization. The implanted constructs together with surrounding tissues were collected for histological examination, and images were acquired at 20× magnification. Quantification of angiogenesis was performed by manual counting of CD31-positive vessels in six randomly selected fields per group. Investigators were blinded to group assignments during the analysis of both in vitro and in vivo experiments.

### 2.10. TAA-Induced Liver Fibrosis Mouse Model

Liver fibrosis was established in six-week-old Balb/c nude mice by administering thioacetamide (TAA; Sigma-Aldrich) intraperitoneally at a dose of 100 mg/kg, three times per week for four consecutive weeks. The establishment of fibrosis was confirmed histologically using H and E and Picrosirius red staining of liver sections. After fibrosis induction, mice were anesthetized by intraperitoneal injection of Alfaxan (30 mg/kg) and Domitor (0.5 mg/kg) following aseptic preparation. A midline laparotomy was performed, and 10 mm recellularized liver scaffold (RLS) pieces from each experimental group were implanted into the interlobular space between the median and right lateral lobes. Postoperative analgesia was provided by subcutaneous administration of meloxicam (5 mg/kg) once daily for three consecutive days. All mice were maintained under standard environmental conditions and randomly assigned to each group. Five mice were assigned purely by chance (Sham, Recellularized, and LXW7-Recellularized), with a total of three groups included in the study. The Sham group served as a surgical control and underwent laparotomy without scaffold implantation. To sustain the fibrotic microenvironment, mice continued to receive TAA (100 mg/kg, IP) twice weekly for an additional four weeks following implantation. At four weeks post-transplantation, mice were humanely euthanized using a lethal dose of anesthesia, and host livers adjacent to the implanted constructs were collected for histological evaluation (H and E, Picrosirius Red) and IF staining for albumin and transforming growth factor-β1 (TGF-β1). For RNA isolation and Reverse Transcription Quantitative Polymerase Chain Reaction (RT-qPCR), host liver tissues surrounding the implantation site were collected to assess fibrosis-related gene expression. Total RNA was extracted using the RNeasy^®^ Mini kit (Qiagen, Hilden, Germany), and RNA purity and concentration were determined using spectrophotometry. Subsequently, 1 µg of total RNA was reverse transcribed into cDNA using the TOPscriptTM RT DryMix (dN6Plus, Enzynomics, Daejeon, Republic of Korea). The relative expression levels of target genes were analyzed using the ^ΔΔ^Ct method, with GAPDH serving as the internal normalization control. Custom primers were sourced from the SFC, Cheongju, Korea, and their sequences are listed in Table 1. Serum samples were collected prior to implantation and at the end point to assess liver function, alanine aminotransferase (ALT), and aspartate aminotransferase (AST) levels measured on a Beckman Coulter AU480 Chemistry Analyzer (Brea, CA, USA).

### 2.11. Immunohistochemical and Immunofluorescence Staining

IHC was performed using the ABC Detection IHC Kit (Abcam) according to the manufacturer’s instructions. Paraffin-embedded sections were deparaffinized, rehydrated, and subjected to antigen retrieval, followed by incubation with primary antibodies: anti-Collagen IV (1:100, ab6586, Abcam), Laminin (PA1-16730, Invitrogen, Waltham, MA, USA), anti-Fibronectin (ab268020, Abcam), anti-CD31 (1:500, ab18298, Abcam), and Ki-67 (1:150, MA5-14520, Invitrogen). For IF staining, sections were permeabilized with 0.1% Triton X-100 for 15 min and blocked with 2% bovine serum albumin (Sigma-Aldrich) for 45 min. Tissue sections were incubated overnight with primary antibodies including anti-human CD31 (1:100, MA5-13188, Invitrogen), Albumin (1:200, PA5-89332, Invitrogen), anti-Integrin αIIb (1:100, sc-21783, Santa Cruz Biotechnology, Dallas, TX, USA), and anti-TGF-β1 (1:200, ab170874, Abcam). After washing, fluorescent dye-conjugated secondary antibodies, Goat anti-Mouse IgG (H+L) Cross-adsorbed, Texas Red-X (Invitrogen), and goat anti-mouse IgG (1:1000, Invitrogen) were applied for one hour, followed by nuclear staining with DAPI. Imaging was performed using an Optinity KCS3-50SS microscope.

### 2.12. Statistical Analysis

Statistical analyses were carried out using Microsoft Excel (Microsoft Corp., Washington, DC, USA), while graphs and plots were generated using OriginPro 10.1.5.132 (OriginLab Corp., Northampton, MA, USA). Quantitative data are presented as the mean ± standard deviation (SD) unless otherwise specified. Statistical comparisons between two groups were performed using Student’s *t*-test, while comparisons among multiple groups were analyzed using one-way (ANOVA) followed by Tukey’s post hoc test. A *p*-value of less than 0.05 was considered to indicate statistical significance.

## 3. Results

### 3.1. In Vitro Evaluation of Endothelial Cell Adhesion Properties of LXW7

**Figure 1:**
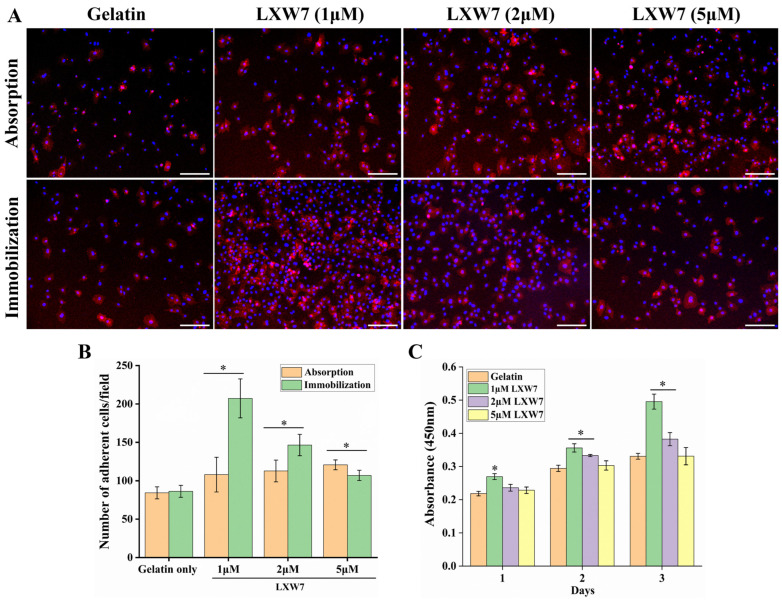
Evaluation of LXW7-mediated HUVECs adhesion. (**A**) Representative fluorescence images of HUVECs (CD31, red) cultured on gelatin alone or gelatin functionalized with LXW7 at different concentrations (1, 2, and 5 μM) via absorption or covalent immobilization. Scale bar = 200 μm. (**B**) Quantification of adherent HUVECs per microscopic field demonstrates significantly higher adhesion on LXW7 immobilized substrates. (**C**) CCK-8 assay results showing increased cell proliferation on LXW7 modified surfaces over 3 days, with the highest proliferation observed on 1 μM immobilized LXW7. Data are presented as mean ± SD, *n* = 3. (* *p* < 0.05 vs. control).

To evaluate the binding affinity of LXW7 toward HUVECs, different concentrations of LXW7 (1, 2, and 5 μM) were immobilized on gelatin-coated plates and assessed for cell attachment. HUVECs were seeded onto gelatin surfaces conjugated with LXW7 either by absorption or covalent immobilization and allowed to adhere for 2 h. IF staining with CD31 and quantification of DAPI-stained cells confirmed significantly higher attachment of HUVECs on surfaces immobilized with 1 μM LXW7 compared to both untreated gelatin and LXW7 adsorption groups (Figure 1A,B). These findings were consistent with the results obtained from crystal violet staining, further confirming the enhanced cell adhesion on LXW7 functionalized surfaces (Supplementary Figure S1). Immobilization ensures more stable and durable binding of LXW7 to the surface, thereby providing superior ligand presentation for endothelial adhesion. Furthermore, CCK-8 assay results over a 3-day culture period demonstrated a dose-dependent effect of LXW7 on HUVEC proliferation, with 1 μM LXW7 showing significantly higher cell proliferation compared to gelatin control and higher LXW7 concentrations. HUVECs attachment was markedly enhanced at 1 μM LXW7; however, increasing the concentration to 5 μM resulted in reduced cell adhesion. This reduction is likely due to saturation and steric hindrance effects at the cell–material interface. At elevated peptide densities, excessive LXW7 presentation may disrupt optimal integrin binding and clustering, thereby limiting focal adhesion formation. Additionally, high surface peptide density may mask native ECM ligands or alter surface conformation, further reducing overall cell adhesion efficiency. Among the tested concentrations, 1 μM LXW7 showed a statistically significant enhancement in both HUVEC adhesion and CCK-8 proliferation compared with other LXW7 concentrations and controls and was therefore identified as the optimal condition for subsequent experiments, as confirmed by one-way ANOVA with post hoc multiple-comparison tests. Collectively, these findings demonstrate that 1 μM LXW7 immobilization markedly enhances HUVECs attachment on gelatin-coated surfaces.

### 3.2. Characterization of Decellularized Rat Liver

**Figure 2:**
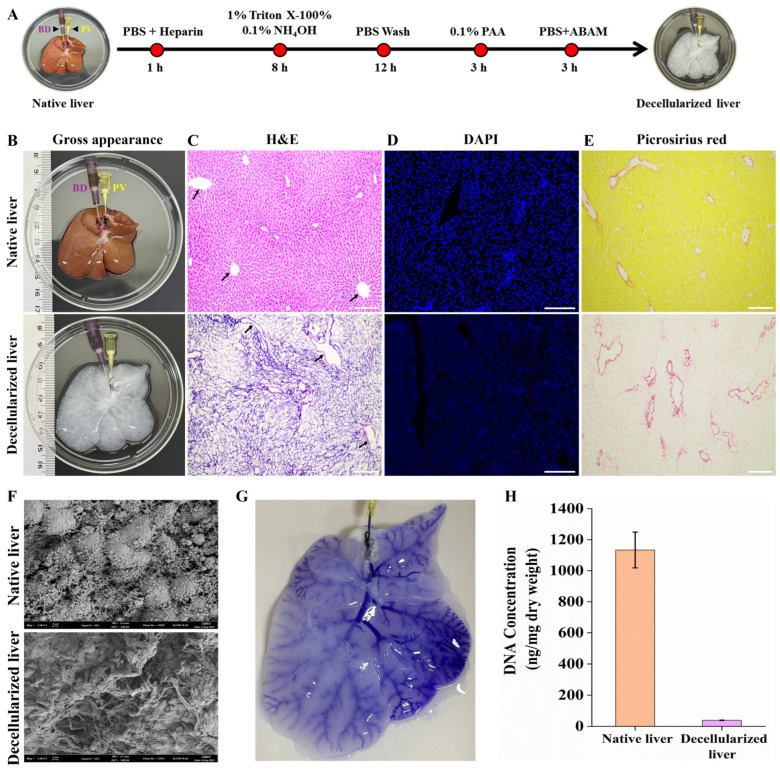
Decellularization of rat liver. (**A**) Schematic diagram showing the strategy for the decellularization of the rat liver. (**B**) Gross morphology of the decellularized liver appears translucent, with a white color and preserved anatomical structure. (**C**,**D**) H and E and DAPI staining confirm complete removal of cellular components, with arrows indicating blood vessels. Scale bar = 200 µm. (**E**) Picrosirius red staining of DLS confirms the preservation of collagen. Scale bar = 200 µm (**F**) SEM analysis of DLS demonstrates the absence of residual cells and intact ECM ultrastructure. Scale bar = 2 µm. (**G**) Crystal violet perfusion via the PV shows the intact vascular tree of the DLS. Scale bar = 2 cm. (**H**) Quantitative DNA analysis of native and decellularized liver.

The rat liver decellularization was successfully achieved by perfusing 1% Triton X-100 and 0.1% ammonium hydroxide through the PV (Figure 2A). During the process, the liver gradually changed in appearance from light brown to translucent white, while retaining the gross morphology of the native liver upon complete decellularization (Figure 2B). Histological analyses with H and E and DAPI staining confirmed complete removal of cellular and nuclear material, with preservation of the ECM architecture (Figure 2C,D). Picrosirius red staining further demonstrated the retention of collagen fibers (Figure 2E), whereas SEM revealed an intact ECM ultrastructure and absence of residual cells (Figure 2F). Perfusion with crystal violet dye via the PV highlighted the intact vascular tree, demonstrating preservation of the vascular architecture and scaffold perfusability (Figure 2G). Quantitative DNA analysis showed that the DNA content of the DLS was reduced to <50 ng/mg tissue, confirming efficient removal of nuclear material (Figure 2H). Furthermore, IHC staining for key ECM components, collagen IV, laminin, and fibronectin, confirmed that the DLS retained its essential ECM architecture (Supplementary Figure S2). Collectively, these findings demonstrate the effectiveness of the decellularization protocol and confirm that the resulting DLS preserves critical ECM components and vascular integrity, thereby providing a suitable platform for subsequent recellularization and liver tissue engineering applications.

### 3.3. Characterization of LXW7 Immobilized DLS

**Figure 3:**
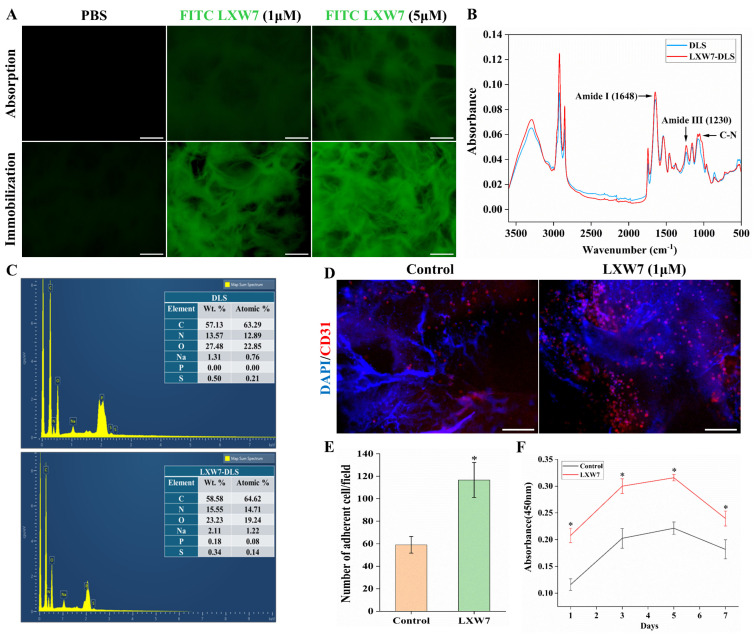
Characterization of LXW7 immobilized DLS. (**A**) Fluorescence microscopy of FITC-labeled LXW7 immobilized on DLS, confirming efficient immobilization. Scale bar = 200 µm. (**B**) FTIR spectra of DLS and LXW7-DLS showing characteristic peaks corresponding to amide I, amide III, and C–N stretching. (**C**) EDS spectra and elemental composition tables demonstrate increased nitrogen content in LXW7-DLS compared to DLS. (**D**) IF staining of HUVECs (CD31, red; DAPI, blue) showing enhanced attachment on LXW7 modified scaffolds compared to controls. Scale bar = 100 µm. (**E**) Quantification of adherent cells per microscopic field showing significantly greater adhesion on LXW7-DLS. (**F**) CCK-8 assay demonstrating enhanced cell proliferation on LXW7-DLS over a 7-day culture period compared to control scaffolds (*n* = 3, * *p* < 0.05).

The successful immobilization of the LXW7 peptide onto DLS was confirmed by fluorescence imaging using FITC-labeled LXW7, FTIR analysis, and elemental mapping. Fluorescence microscopy showed that LXW7 conjugated DLS exhibited markedly higher fluorescence intensity compared to unconjugated scaffolds (Figure 3A). Importantly, the immobilization method provided superior peptide retention and stability over the simple absorption method, ensuring a more durable and uniform presentation of LXW7 on the scaffold surface. Furthermore, the FTIR spectra of unmodified DLS and LXW7-DLS are presented in Figure 3B. The overall spectral profiles of both groups were largely similar. However, compared with DLS, LXW7-DLS showed an obvious increase in the intensity of the amide I band (~1648 cm^−1^), along with enhanced signals in the amide III (~1230 cm^−1^) and C–N stretching regions. These changes indicate an increased contribution of peptide-derived amide groups following LXW7 modification and provide supportive evidence for successful immobilization of LXW7 on the scaffold surface. EDS analysis revealed an increased nitrogen content in LXW7-DLS compared with unmodified DLS (Figure 3C), which is consistent with the presence of peptide modification on the scaffold surface. Taken together, the fluorescence and FTIR results, with EDS serving as complementary information, indicate successful LXW7 functionalization of the DLS.

Functionally, LXW7 immobilization significantly enhanced HUVECs attachment, proliferation, and inhibited platelets adhesion on LXW7-DLS pieces. IF staining for CD31 revealed that 1 μM LXW7 immobilized scaffolds supported greater HUVECs attachment compared to unmodified control (Figure 3D). Quantification of DAPI-stained nuclei confirmed a marked increase in adherent cells on the LXW7 immobilized scaffold (Figure 3E). Furthermore, the CCK-8 assay demonstrated that LXW7-modified scaffolds promoted significantly higher cell proliferation over a 7-day culture period relative to the control (Figure 3F). Platelet adhesion assays further demonstrated that LXW7-coated gelatin plates and LXW7-modified DLS scaffolds exhibited markedly reduced platelet adhesions compared to unmodified groups, underscoring the antiplatelet property of LXW7 (Supplementary Figure S3). Altogether, these findings indicate that LXW7 immobilization improves HUVECs adhesion and proliferation while simultaneously reducing platelet adhesion, thereby validating its functional contribution to enhancing scaffold endothelialization and hemocompatibility.

### 3.4. Efficient Re-Endothelialization of the LXW7 Modified DLS

**Figure 4:**
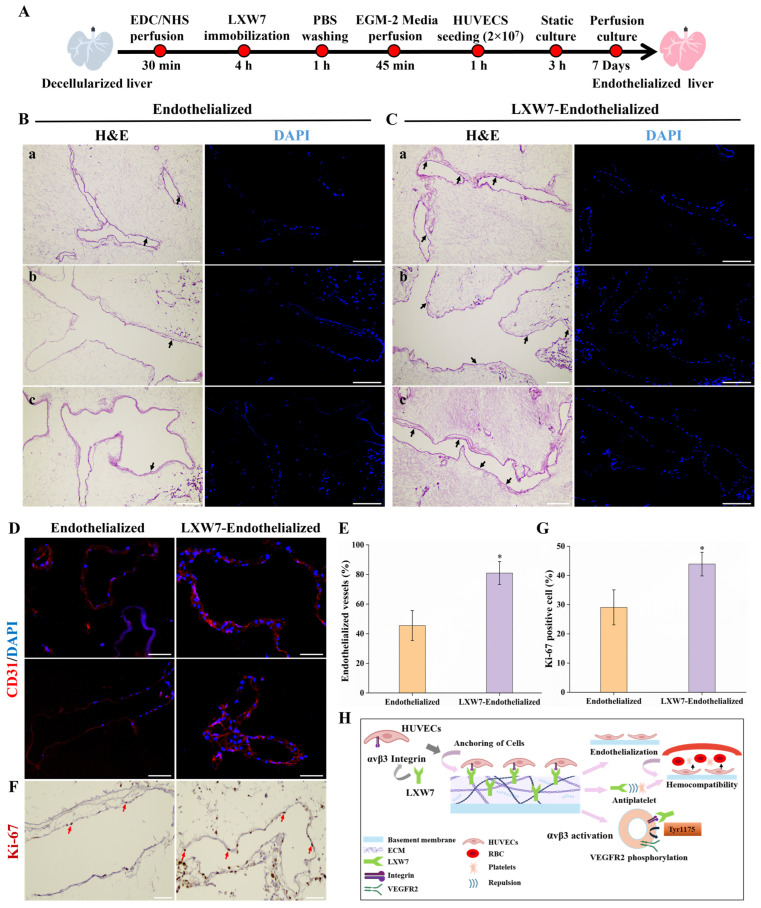
Re-endothelialization of DLS using HUVECs. (**A**) Schematic diagram illustrating the re-endothelialization protocol. (**B**,**C**) H and E and DAPI staining of 3 different lobes (**a**). Left lateral, (**b**). Median, (**c**). Right lateral of endothelialized and LXW7 endothelialized scaffold. LXW7 endothelialized scaffold showing a well-spread monolayer of HUVECs adhered to the vessels (black arrows), while poor cell distribution in the unmodified group. Scale bar = 200 μm. (**D**) IF staining for CD31 (red) and DAPI (blue) demonstrates improved endothelial lining in LXW7 endothelialized scaffolds compared to controls. Scale bar = 50 µm. (**E**) Quantitative analysis of endothelialized vessels indicates significantly greater coverage in LXW7 endothelialized scaffolds (*n* = 3 field/lobe). (**F**) Ki-67 immunostaining (red arrows) showing proliferating HUVECs within the vasculature. (**G**) Quantification of Ki-67 positive cells *(n* = 3 field/lobe), (* *p* < 0.05 vs. endothelialized). (**H**) Mechanisms of LXW7 in the re-endothelialization of the scaffold.

In our study, DLS were immobilized with the LXW7 peptide, an integrin αvβ3-specific ligand, to enhance HUVECs adhesion, proliferation, and re-endothelialization. LXW7 immobilization provided stable peptide retention on the scaffold surface, ensuring stronger HUVECs capture compared to unmodified scaffolds. Following immobilization, HUVECs were seeded onto the scaffolds and cultured for seven days (Figure 4A). After 7 days of perfusion culture, constructs were retrieved and evaluated using histological and functional assays. H and E and DAPI staining demonstrated that LXW7 immobilized endothelialized scaffolds supported strong HUVECs adhesion along the vasculature, forming a continuous and well-defined endothelial lining. In contrast, unmodified scaffolds showed poor vascular coverage and irregular cell distribution (Figure 4B,C). For an overview of the endothelialized vessels of both groups, H and E staining images of different lobes are provided in Supplementary Figure S4. IF staining for CD31 further confirmed enhanced HUVECs lining within the vasculature of LXW7 endothelialized scaffolds compared to endothelialized scaffolds only (Figure 4D). Quantitative analysis revealed that approximately 81 ± 8% of the vasculature in LXW7 scaffolds was covered by HUVECs, compared with only 45 ± 10% in unmodified scaffolds (Figure 4E). In addition, Ki-67 staining demonstrated increased numbers of proliferating cells in the LXW7 endothelialized group, which was further supported by quantification showing a significantly greater proportion of Ki-67 positive cells (Figure 4F,G). LXW7 enhances endothelialization of DLS by binding to αvβ3 integrins of HUVECs, thereby promoting cell adhesion and proliferative signaling. This integrin–mediated interaction results in more continuous endothelial coverage, reduced platelet adhesion, and overall improved hemocompatibility within the scaffold (Figure 4H).

**Figure 5:**
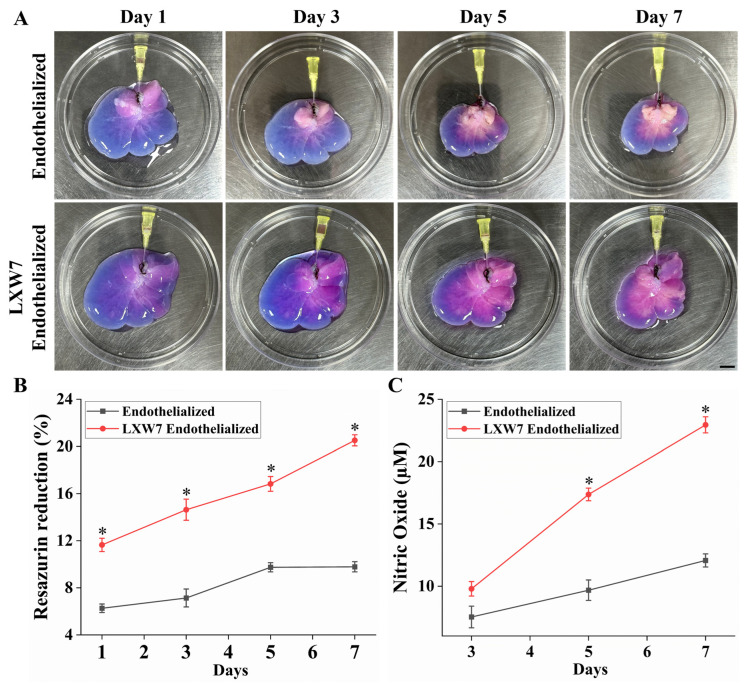
Functional assessment of endothelialized scaffolds. (**A**) Representative images of the resazurin reduction assay performed on endothelialized and LXW7 endothelialized scaffolds over a 7-day culture period. LXW7 endothelialized scaffolds exhibited a more pronounced color change, indicating enhanced metabolic activity and cell proliferation. scale bar = 2 cm. (**B**) Quantitative analysis demonstrated a significantly higher percentage of resazurin reduction in LXW7 endothelialized scaffolds compared to controls over time. (**C**) Nitric oxide production was also significantly elevated in LXW7 endothelialized scaffolds, further confirming enhanced endothelial function (*n* = 3 scaffold * *p* < 0.05 vs. Endothelialized).

In our study, a resazurin reduction perfusion assay was conducted to evaluate the viability and proliferative activity of HUVECs reseeded onto the liver scaffolds. A resazurin medium working solution was perfused through the scaffolds at a rate of 2 mL/min for 1 h. LXW7 endothelialized scaffolds displayed a more intense change each day of perfusion, indicating enhanced HUVECs attachment and proliferation (Figure 5A). Quantitative analysis further confirmed a significantly higher percentage reduction in resazurin in LXW7 immobilized endothelialized scaffolds compared to endothelialized only, reflecting accelerated cell proliferation over the 7-day culture period (Figure 5B). In contrast, unmodified endothelialized scaffolds demonstrated lower resazurin reduction, with a slight decline by day 7, suggesting reduced proliferative activity. Nitric oxide release assays showed that LXW7 endothelialized scaffolds exhibited the greatest and most sustained endothelial functional activity throughout the 7-day culture period (Figure 5C). Altogether, these findings demonstrate that LXW7 immobilization promotes the formation of a uniform and functional endothelial lining within the scaffold vasculature, markedly enhancing HUVECs attachment, coverage, and proliferation, thereby significantly improving scaffold re-endothelialization.

### 3.5. Thrombogenicity of Re-Endothelialized Constructs After Porcine Blood Perfusion

**Figure 6:**
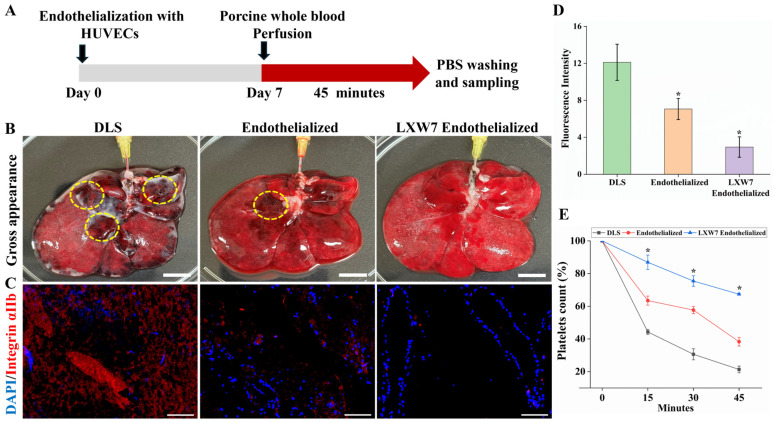
Thrombogenicity evaluation of endothelialized scaffolds (**A**) schematic of the experimental design of ex vivo blood perfusion. (**B**) Gross appearance of DLS, endothelialized, and LXW7 endothelialized scaffolds after blood perfusion, showing markedly reduced clot deposition in the LXW7 group (yellow circles highlight thrombi). Scale bar = 2 cm. (**C**) IF staining of platelet marker integrin αIIb (red) with DAPI (blue) showing reduced platelet adhesion in endothelialized and LXW7endothelialized scaffolds compared to DLS. Scale bar = 100 µm. (**D**) Quantification of fluorescence intensity confirming significantly decreased platelet adhesion in LXW7 endothelialized scaffolds (*n* = 4 fields, * *p* < 0.05 vs. DLS). (**E**) Time-dependent changes in platelet count (%) in the blood perfusate during ex vivo blood perfusion (*n* = 3 samples each time point, * *p* < 0.05 vs. DLS).

To evaluate the thrombogenicity of DLS, endothelialized and LXW7 endothelialized scaffolds were subjected to ex vivo perfusion with sodium citrate anticoagulant porcine whole blood for 45 min following 7 days of HUVECs culture (Figure 6A). Gross observation revealed that unmodified DLS accumulated visible thrombi within vascular channels, whereas endothelialized scaffolds showed reduced clot formation. In contrast, LXW7 endothelialized scaffolds exhibited minimal clot deposition, indicating superior antithrombogenic properties (Figure 6B). IF staining for integrin αIIb confirmed markedly reduced platelet adhesion in LXW7 endothelialized scaffolds compared to DLS and endothelialized scaffolds (Figure 6C). Quantification of fluorescence intensity further supported these observations, showing significantly lower platelet adhesion in the LXW7 group, highlighting the antiplatelet adhesion properties of LXW7 (Figure 6D). Furthermore, in the decellularized and uncoated endothelialized group, a marked decrease in platelet count was observed in the perfused blood compared to LXW7 endothelialized at each time point, reflecting superior endothelial integrity and enhanced blood compatibility (Figure 6E). Collectively, these findings indicate that LXW7 immobilization enhances hemocompatibility by reducing thrombus formation and platelet adhesion.

### 3.6. Co-Culture of HepG2 Cells and HUVECs on DLS

**Figure 7:**
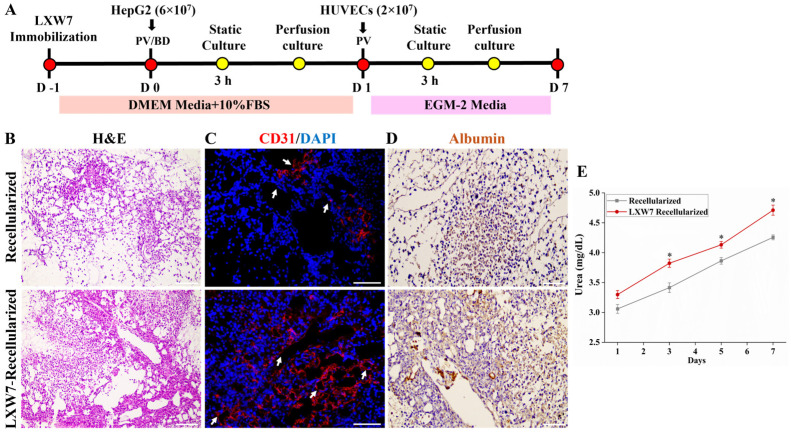
Co-culture of HepG2 and HUVECs. (**A**) Schematic diagram illustrating the co-culture protocol. (**B**) H and E staining of LXW7 recellularized scaffolds showing HepG2 cells localized within the parenchymal regions, while HUVECs were aligned along the vascular walls. scale bar = 200 μm. (**C**) IF staining with CD31 demonstrates HUVECs localization along the vascular channels, forming endothelial linings (white arrows). scale bar = 100 μm. (**D**) IHC for albumin (brown) demonstrates the presence of HepG2 in the parenchyma. Scale bar = 100 µm. (**E**) Urea production assay showing significantly higher secretion in LXW7 recellularized scaffolds compared to recellularized scaffolds over 7 days (* *p* < 0.05).

To investigate the impact of LXW7-DLS on hepatocyte and ECs co-recellularization, scaffolds were seeded with HepG2 cells and HUVECs and maintained under perfusion culture for 7 days (Figure 7A). For spatial compartmentalization, HepG2 cells were introduced through both the PV and bile duct to populate the parenchymal regions, followed by HUVECs seeding via the PV to reline the vascular lumens. H and E staining confirmed widespread cellular infiltration throughout the parenchymal regions in both groups, with the LXW7 modified DLS showing more uniform and denser repopulation of hepatic lobule-like areas (Figure 7B). Further IF staining for CD31 showed that HUVECs localized along the vascular channels, forming distinct endothelial linings, while HepG2 cells were distributed throughout the parenchymal compartments, filling the lobule-like spaces. Importantly, LXW7 recellularized scaffolds exhibited markedly enhanced endothelial coverage together with denser and more uniform HepG2 repopulation, reflecting more effective re-endothelialization and improved hepatocyte integration (Figure 7C). Consistently, IHC staining for albumin revealed stronger and more extensive hepatocyte functional protein expression in the LXW7 group, with hepatocytes widely distributed across the parenchymal compartments. In contrast, unmodified scaffolds showed fewer albumin-positive cells and weaker hepatocyte integration, indicating that LXW7 modification enhances hepatocyte function and metabolic activity (Figure 7D). Functional analysis using a urea secretion assay showed a progressive increase in urea production from days 1 to 7 in recellularized scaffolds; however, LXW7 modified recellularized scaffolds consistently exhibited significantly higher urea production, indicating superior hepatic metabolic function (Figure 7E). Altogether, the co-culture of HepG2 cells with HUVECs enhances hepatocyte engraftment, phenotype maintenance, and hepatic function within co-cultured scaffolds, thereby establishing a more physiologically relevant microenvironment.

### 3.7. Evaluation of Angiogenic Potential of Modified Scaffolds In Vivo

**Figure 8:**
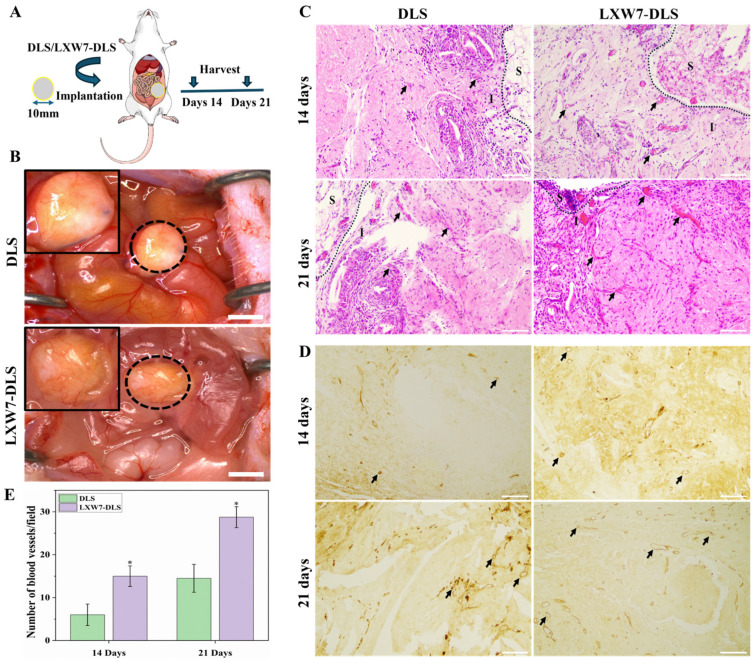
In vivo angiogenesis assessment following DLS implantation into the mouse omentum. (**A**) Schematic of experimental design showing implantation of DLS, LXW7-DLS into the omentum of mice. (**B**) Gross images of implants demonstrate greater vascularization into LXW7-DLS compared to DLS (Black circles highlight implant scaffold; black boxes indicate the magnified image of the implant area). Scale bar = 2 cm. (**C**,**D**) H and E staining and IHC showing increased infiltration of blood vessels (black arrows) in LXW7-DLS compared to DLS at 14 and 21 days (The black dashed line indicates the boundary separating the surrounding tissue (S) from the implanted scaffold area (I)). Scale bar = 100 µm. (**E**) Quantitative analysis of vessel density per microscopic field, showing significantly greater blood vessel formation in LXW7-DLS at 14 and 21 days (* *p* < 0.05 vs. DLS).

The angiogenic potential of LXW7 modified scaffolds was evaluated following implantation into the omentum of mice (Figure 8A). The scaffolds were harvested at 14 and 21 days of post-implantation. The mice showed no signs of inflammation or infection, indicating a good biocompatibility of the implanted scaffold. Gross evaluation at 21 days post-implantation demonstrated that LXW7 modified DLS exhibited markedly greater neovascular formation than the unmodified scaffolds (Figure 8B). H and E staining demonstrated a higher density of infiltrating blood vessels in LXW7-DLS scaffolds, with more mature vascular structures observed at 21 days (Figure 8C). IHC staining for CD31 further confirmed increased neovascularization in LXW7-DLS compared to DLS at both 14 and 21 days (Figure 8D). Quantitative analysis showed a significant increase in the number of blood vessels per field in LXW7-DLS compared to DLS at both time points (Figure 8E). These results indicate that LXW7-modified DLS markedly enhances the recruitment of host EC, leading to robust vascularization of scaffolds in vivo.

### 3.8. In Vivo Evaluation of Recellularized Scaffolds in a TAA-Induced Fibrotic Liver Model

**Figure 9:**
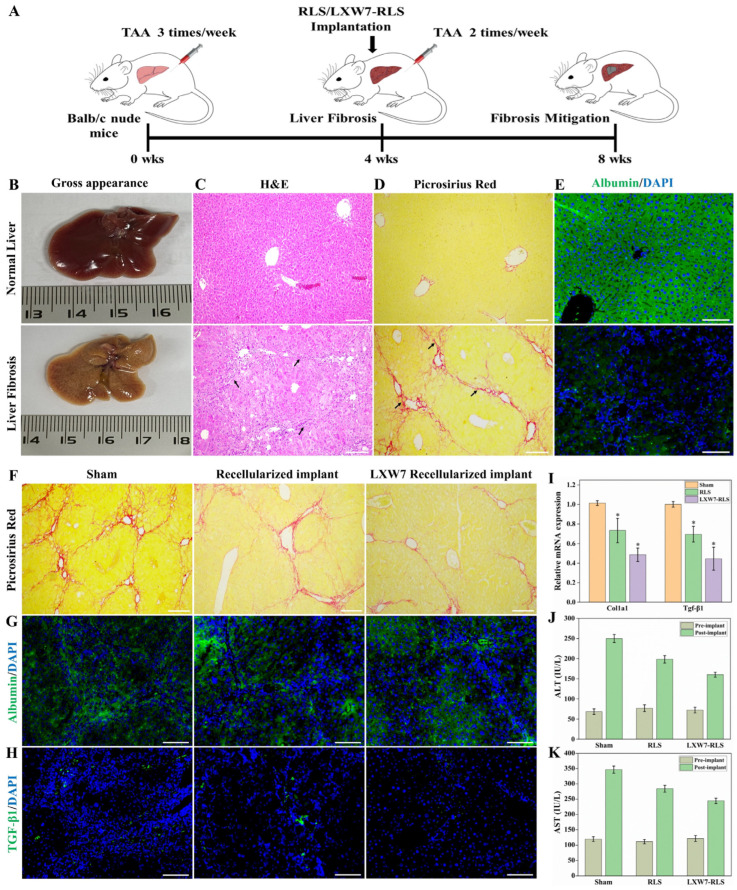
TAA-induced liver fibrosis mouse model. (**A**) Schematic of the experimental design showing TAA-induced fibrosis, scaffold implantation, and harvest timeline. (**B**) Gross images of normal and fibrotic mouse liver. (**C**,**D**) H and E and Picrosirius red staining showing distorted hepatic architecture and collagen accumulation in fibrotic livers, arrow indicates bridging fibrosis. Scale bars = 200 µm. (**E**) IF staining for albumin (green) and DAPI (blue) shows reduced hepatic function in fibrosis. Scale bars = 100 µm. (**F**) Picrosirius red staining of sham, RLS, and LXW7 RLS implants showing reduced collagen deposition. Scale bars = 200 µm. (**G**,**H**) IF for albumin and TGF-β1 demonstrating enhanced hepatocyte function and reduced fibrogenic signaling in LXW7 RLS implants. Scale bars = 100 µm. (**I**) qPCR analysis showing decreased expression of fibrosis markers (Col1a1, TGF-β1) in the LXW7 group (* *p* < 0.05 vs. Sham). (**J**,**K**) Serum ALT and AST levels were significantly reduced in the LXW7 group compared to controls.

A TAA-induced liver fibrosis mouse model was established to assess the therapeutic efficacy of recellularized liver scaffolds (RLS) (Figure 9A). TAA administration for 4 weeks resulted in the development of liver fibrosis in mice, as evidenced by gross and histological changes in the liver. Gross examination revealed that fibrotic livers were shrunken and nodular compared to normal livers (Figure 9B). Histological analysis with H and E staining demonstrated severe architectural distortion, hepatocyte degeneration, and bridging fibrosis, while Picrosirius red staining confirmed extensive collagen deposition in fibrotic livers (Figure 9C,D). IF staining of albumin further showed reduced albumin expression in fibrotic livers (Figure 9E). This was accompanied by a significant reduction in body weight gain throughout the TAA induction period (Supplementary Figure S5), validating the successful establishment of a fibrotic model.

Following implantation in the liver fibrosis mouse model, RLS and LXW7-RLS exhibited a marked reduction in collagen deposition in host liver tissue compared to sham (Figure 9F). IF analysis showed higher albumin expression in the livers of mice implanted with LXW7 modified RLS scaffolds, indicating improved hepatocyte function and mitigation of liver fibrosis (Figure 9G). This was accompanied by reduced TGF-β1 expression, demonstrating attenuation of fibrogenic signaling in the LXW7 group (Figure 9H). Consistently, qPCR analysis revealed downregulation of fibrosis-associated genes, Col1a1 and TGF-β1, in host liver tissue implanted with LXW7 RLS relative to RLS and sham (Figure 9I). Furthermore, serum biochemical assays confirmed that ALT and AST levels were significantly lower in mice implanted with LXW7 recellularized compared to Sham (Figure 9J,K). Overall, these findings demonstrate that recellularized liver scaffolds, particularly LXW7-RLS, mitigate fibrosis progression and restore hepatocyte function.

## 4. Discussion

Tissue engineering has rapidly advanced as a key area of regenerative medicine, with decellularized scaffolds gaining particular attention in liver engineering. Recent progress in decellularization techniques has enabled the creation of organ-derived ECM constructs that preserve native architecture and biochemical cues, moving this approach from experimental studies toward clinical translation [31]. However, the decellularization process inherently removes the native endothelial lining, leaving the vascular channels exposed and prone to thrombosis and inflammatory cell adhesion. This loss of endothelium represents a major barrier to clinical application, as the vascular lining normally functions as a dynamic, non-adhesive barrier to platelets and leukocytes [32]. Therefore, achieving rapid and stable endothelialization is not only desirable but critical for graft patency, hemocompatibility, and long-term success in scaffold implantation.

In our study, we modified DLS with LXW7, an integrin αvβ3-specific ligand, as a promising strategy to overcome the challenge of endothelialization. LXW7 selectively binds EPCs and ECs, thereby enhancing their adhesion, proliferation, and vascular network formation, while showing minimal affinity for platelets. This selective interaction is particularly advantageous, as it facilitates rapid and stable HUVECs attachment while simultaneously reducing thrombogenic responses. The establishment of a functional endothelial lining is critical not only for improving hemocompatibility but also for providing a supportive vascular microenvironment that fosters hepatocyte engraftment; thus, advancing the development of clinically relevant bioengineered liver constructs. Compared with conventional approaches such as RGD peptide modification or antibody-based coatings, which often suffer from non-specific cell adhesion [16], high platelet affinity [17], high production costs and potential immunogenicity, LXW7 offers a more selective and translationally feasible strategy. By integrating LXW7 with DLS, our approach directly addresses the limitations of earlier methods and represents a significant step forward in engineering functional and hemocompatible liver grafts.

Previous studies have demonstrated that LXW7, an integrin αvβ3-specific ligand, is effective in functionalizing small-diameter vascular grafts, where it enhances EPCs/ECs capture, promotes rapid endothelialization, and reduces platelet adhesion [21,33,34,35]. Mechanistically, LXW7 binds specifically to αvβ3 integrins on ECs, allowing them to firmly attach to the scaffold surface. This targeted binding promotes rapid cell adhesion, spreading, and alignment, enabling the formation of a stable and continuous endothelial layer. LXW7 αvβ3 engagement also activates pro-survival and angiogenic signaling, including VEGFR2 phosphorylation (Tyr1175), which supports endothelial function and vascular maturation. At the same time, LXW7 has minimal interaction with platelet integrins, resulting in reduced platelet adhesion and decreased thrombosis. Through these coordinated effects, LXW7 enhances endothelialization, strengthens vascular stability, and improves hemocompatibility of the scaffold. However, its potential application for re-endothelialization in whole organ bioengineering has not been investigated. For the first time, we have employed LXW7 to functionalize DLS, aiming to promote rapid endothelialization, improve hemocompatibility, and provide a supportive niche for hepatocyte engraftment in the development of bioengineered liver constructs.

First, we validated HUVECs’ attachment on gelatin-coated plates functionalized with LXW7, and subsequently on DLS. Our findings underscore the effectiveness of LXW7 immobilization as a biofunctionalization strategy to promote HUVECs attachment, consistent with earlier studies reporting enhanced EC attachment and proliferation on LXW7 modified vascular grafts [17,21,33]. Although both adsorption and immobilization facilitated peptide presentation; however, covalent immobilization achieved superior ligand stability and markedly enhanced HUVECs adhesion. Importantly, 1 µM LXW7 was identified as the optimal concentration, as higher densities of 5 µM disrupted integrin ligand interactions, leading to reduced cell attachment. These observations highlight the critical role of crosslinking chemistry in ensuring durable and biologically functional peptide presentation [27], which is essential for establishing stable endothelialization and advancing scaffold-based liver bioengineering toward clinical translation.

At the same time, the success of re-endothelialization also depends on the preservation of ECM integrity during decellularization. While decellularization is necessary to remove cellular and immunogenic components, it can inadvertently damage key ECM constituents such as fibronectin, laminin, and collagen IV, which are essential for ECs’ adhesion, polarity, and survival [36]. Disruption of basement membrane architecture and alterations in biomechanical properties further compromise cytoskeletal organization and barrier formation, thereby limiting effective endothelialization [37,38]. To address this, we employed a relatively gentle decellularization protocol using Triton X and ammonium hydroxide, instead of harsh ionic detergents such as SDS, in order to minimize ECM damage and preserve structural and biochemical cues [39].

Our study demonstrates that LXW7 immobilization on DLS significantly improves re-endothelialization efficiency and hemocompatibility. LXW7 modified scaffolds supported the formation of a continuous and functional endothelial lining. This outcome is consistent with prior studies on small-diameter vascular grafts, where LXW7 demonstrated strong endothelial selectivity and low platelet affinity. The ability of LXW7 to sustain endothelial viability and nitric oxide activity over extended culture suggests that it provides not only a structural lining but also functionally active endothelium, which is essential for long-term graft patency. Moreover, the reduced thrombogenicity observed in blood perfusion experiments underscores the translational relevance of this approach, as thrombus formation and platelet adhesion remain key obstacles in vascularized organ scaffolds. This antithrombogenic effect is likely attributable to the dual action of LXW7, its ability to promote the formation of a functional endothelium while simultaneously exerting antiplatelet activity.

Additionally, our study underscores the role of LXW7-mediated endothelialization in enhancing hepatocyte engraftment and function within RLS. The increased amount of albumin and urea production observed in LXW7 modified scaffolds reflects improved hepatocyte viability. LXW7 immobilization supported the establishment of a continuous endothelial lining, which in turn created a vascular niche that stabilized hepatocyte localization and sustained their metabolic activity. This outcome is consistent with the concept that endothelial parenchymal interactions are essential for hepatocyte survival and function [40]. The factors secreted from vessels, such as NO and VEGF, helped to sustain hepatocytes’ viability and, moreover, improved both the structural and functional maturation of the bioengineered constructs. This is consistent with the previous report demonstrating that NO inhibition led to a significant reduction in HepG2 cluster sizes within bioengineered liver [41]. The enhanced hepatocyte phenotype and function observed in LXW7 modified scaffolds; therefore, highlight the interdependence of vascular and parenchymal compartments in whole liver bioengineering.

The in vivo omentum implantation studies highlight that LXW7 modification markedly improves biocompatibility and the angiogenic response. LXW7-DLS supported enhanced recruitment of host EC, increased vessel formation, and maturation. These findings are consistent with an earlier study in skin tissue engineering [42], where LXW7 was shown to recruit and retain EPCs through αvβ3 engagement and VEGFR2 activation [17], thereby sustaining angiogenic function under ischemic conditions. Therefore, LXW7 modification not only accelerates biocompatibility but also provides a microenvironment capable of sustaining angiogenic function, thereby advancing scaffold performance toward translational applications.

The LXW7-RLS constructs exhibited functional capacity to provide both structural support and paracrine signals that modulate the repair process. Heterotopic implantation of RLS and LXW7 recellularized scaffolds alleviated fibrotic changes and improved liver function in the fibrotic mouse liver. The observed reduction in collagen deposition, improved albumin expression, and lower serum ALT/AST levels collectively indicate that LXW7 functionalization promotes hepatocyte activity while attenuating fibrogenic signaling. These beneficial effects can be attributed to the recellularized scaffold, which generates biochemical cues that support tissue repair and regeneration. The presence of both endothelial and parenchymal cells within the constructs further contributed to functional integration and mitigated the liver fibrosis progression. The reduction in fibrosis observed in the TAA-induced fibrotic liver model likely reflects the combined contribution of both improved endothelialization and restored hepatocyte function. LXW7-mediated endothelialization enhances vascular patency, which improves oxygenation and nutrient delivery within the regenerating tissue. A stable and functional endothelial layer also suppresses platelet activation and inflammatory cell infiltration. Simultaneously, improved hepatocyte viability and function within LXW7-DLS promote metabolic recovery and reduce the secretion of pro-fibrotic cytokines. Together, these effects create a microenvironment that downregulates fibrogenic signaling pathways while supporting healthy parenchymal regeneration, thereby contributing to the overall attenuation of fibrosis. Previous studies have shown that human-derived cells can exert therapeutic effects in rodent models of TAA-induced liver injury [40,43]. However, the use of a short-term mouse fibrosis model may not fully recapitulate the chronic progression of human cirrhosis.

This study was designed to evaluate the structural and functional benefits of LXW7 immobilization on DLS, with less emphasis on the underlying molecular mechanisms. Since LXW7 interacts strongly with integrin αvβ3, further work should aim to elucidate the downstream signaling pathways that regulate re-endothelialization, which may provide valuable mechanistic insights. While LXW7 treatment effectively promoted endothelialization, the study was limited by using simplified cell models such as HepG2 and HUVECs, which cannot fully replicate the behavior of primary hepatocytes or autologous ECs. In this study, HepG2 cells were used as an alternative to primary hepatocytes due to their ready availability, phenotypic stability, and reproducibility, which enabled consistent evaluation of scaffold–cell interactions. However, it is acknowledged that HepG2 cells do not fully recapitulate the metabolic complexity and functional heterogeneity of primary human hepatocytes.

Future studies will therefore focus on the use of iPSC-derived hepatic cells and ECs on scaling the approach in large animal transplantation models, critical for progressing toward clinical translation. This study shows that LXW7-immobilized DLS enhances endothelialization, minimizes thrombogenicity, and establishes a supportive niche that preserves hepatocyte function. By providing both structural endothelial coverage and sustained functional activity, LXW7 overcomes two major challenges, insufficient endothelialization and platelet-driven thrombosis in liver scaffold engineering. LXW7-mediated endothelialization contributes to the mitigation of liver fibrosis by improving vascular patency, reducing inflammatory activation, and supporting functional hepatic remodeling. These findings offer compelling proof-of-concept for the application of LXW7 in advancing whole organ bioengineering.

## 5. Conclusions

One of the major challenges in liver tissue bioengineering is achieving stable endothelialization and hemocompatibility while simultaneously supporting hepatocyte function. LXW7 immobilization of DLS directly addresses these barriers by providing αvβ3-mediated endothelial specificity, which promotes rapid vascular lining formation and reduces platelet adhesion. The resulting re-endothelialized constructs create a supportive microenvironment for hepatocyte survival and function. Collectively, these findings emphasize the promising role of LXW7 in advancing tissue bioengineering approaches for the development of transplantable, vascularized liver constructs. Future studies evaluating LXW7 functionalized scaffolds in large animal models and long-term transplantation settings will be essential to accelerate their translation toward clinical applications in liver regeneration.

## Figures and Tables

**Table 1:** List of primers used for qRT-PCR.

Primer	Sequences (5′ to 3′)	Tm (°C)
**Mouse TGF-β1**	F-GGAGAGCCCTGGATACCAACT R-TGTGTGTCCAGGCTCCAAAT	60.8
**Mouse Col1a1**	F-ACAGTCGCTTCACCTACAGC R-GGGTGGAGGGAGTTTACACG	60.8
**Mouse GAPDH**	F-GCCTCGTCCCGTAGACAAAA R-CAATCTCCACTTTGCCACTGC	60.8

TGF-β1, Transforming growth factor beta 1; Col1a1, Collagen Type I Alpha 1 Chain; GAPDH, Glyceraldehyde-3-phosphate dehydrogenase transcript variant 1.

## Data Availability

The original contributions presented in the study are included in the article/Supplementary Materials further inquiries can be directed to the corresponding author.

## Supplementary Materials

The following supporting information can be downloaded at: https://www.mdpi.com/article/10.3390/jfb17030122/s1, Method S1: Anti platelets properties of LXW7 immobilized surface; Figure S1: HUVECs adhesion assay on gelatin-coated substrates; Figure S2: Immunohistochemistry of ECM protein; Figure S3: Platelet adhesion assay on gelatin-coated plates and DLS scaffolds; Figure S4: An overview of the Endothelialized vessels, Hand E staining images of different lobes of the endothelialized scaffolds; Figure S5: Body weight of normal and liver fibrotic mice.

## Abbreviations

The following abbreviations are used in this manuscript:

ALT	Alanine Aminotransferase
AST	Aspartate Aminotransferase
CCK-8	Cell counting kit-8
DAPI	4′,6-diamidino-2-phenylindole
DLS	Decellularized Liver Scaffold
ECs	Endothelial Cells
ECM	Extracellular matrices
EDC	Ethyl-3-(3-dimethylaminopropyl) carbodiimide hydrochloride
EGM-2	Endothelial Cell Growth Medium-2
FAF BSA	Fatty Acid-free Bovine Serum Albumin
GAPDH	Glyceraldehyde-3-phosphate Dehydrogenase Transcript Variant 1
HepG2	Hepatocellular carcinoma
H&E	Hematoxylin and Eosin
HUVECs	Human Umbilical Vein Endothelial Cells
IF	Immunofluorescence staining
IHC	Immunohistochemical
MMP-2	Matrix Metalloproteinase 2
NHS	N-hydroxy succinimide
NO	Nitric Oxide
NOS3	Nitric Oxide Synthase 3
PBS	Phosphate-Buffered Saline
PFA	Para formaldehyde
PLSCR1	Phospholipid Scramblase 1
PV	Portal Vein
RT-qPCR	Reverse Transcription Quantitative Polymerase Chain Reaction
SDC-1	Syndecan-1
SEM	Scanning Electron Microscopy
TAA	Thioacetamide
TBXAS	Thromboxane A Synthase
TEVGs	Tissue-engineered Vascular Grafts
THBS1	Thrombospondin 1
TGF-β1	Transforming growth factor Beta-1
