# Supplementary material for: LXW7 Peptide Modification of Acellular Liver Scaffolds Improves Endothelialization and Hemocompatibility in Bioengineered Liver
Source: J Funct Biomater. 2026 Mar 3;17(3):122. doi: 10.3390/jfb17030122 (PMC13027059; doi:10.3390/jfb17030122)
Supplement: Supplementary file 1 [file jfb-17-00122-s001.zip › jfb-4119421-supplementary.pdf]

Supplementary Materials

LXW7 Peptide Modification of Acellular Liver Scaffolds Improves Endothelialization and Hemocompatibility in Bioengineered Liver

Usha Yadav, Chandra J. Yadav, Sadia Afrin, Jun-Yeong Lee, Jihad Kamel, Kyung-Mee Park *
College of Veterinary Medicine, Chungbuk National University, Cheongju 28644, Republic of Korea
** Correspondence: parkkm@cbnu.ac.kr*

Method S1. Antiplatelet properties of the LXW7 immobilized surface.

Platelet Attachment Assay: Platelet-rich plasma (PRP) was prepared by centrifugation of whole rat blood and applied for evaluation of platelet adhesion. For 2D assays, 100 μL of PRP was seeded onto gelatin-coated wells and incubated for 2 h at 37 °C in a humidified atmosphere containing 5% CO_2. Following incubation, the wells were washed three times with PBS to remove non-adherent platelets, and the attached cells were fixed in 10% neutral-buffered formalin for 20 min. For 3D evaluation, small pieces of decellularized liver scaffolds (DLS) were incubated with PRP under identical conditions for 2 h. After incubation, scaffolds were thoroughly rinsed with DPBS to remove unbound platelets and subsequently fixed in 10% formalin. The samples were then dehydrated through a graded ethanol series, sputter-coated with gold, and analyzed by scanning electron microscopy (SEM) to assess platelet adhesion and morphology.

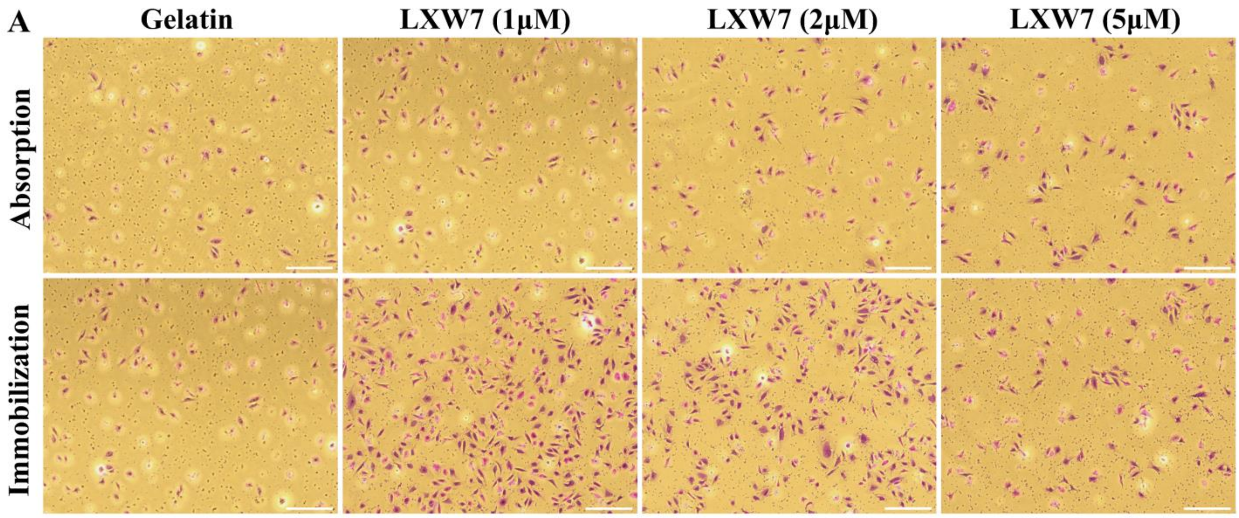

Figure S1. HUVEC adhesion assay on gelatin-coated substrates. (**A**) Representative crystal violet-stained images of HUVECs cultured on gelatin surfaces under different LXW7 conditions. Cell numbers and spreading were markedly increased on LXW7 immobilized surfaces, particularly at 1 μM, indicating enhanced endothelial attachment. Scale bar = 200 μm

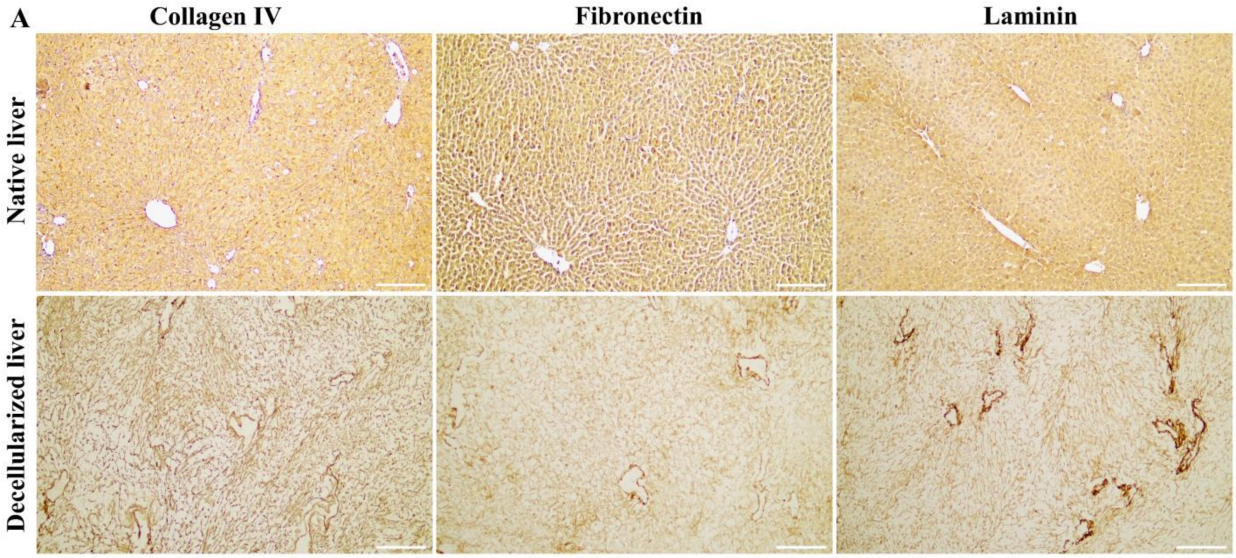

Figure S2. Immunohistochemistry of ECM protein. (**A**) IHC staining of DLS confirming preservation of ECM proteins Collagen IV, Fibronectin, and Laminin. Scale bar = 200 μm.

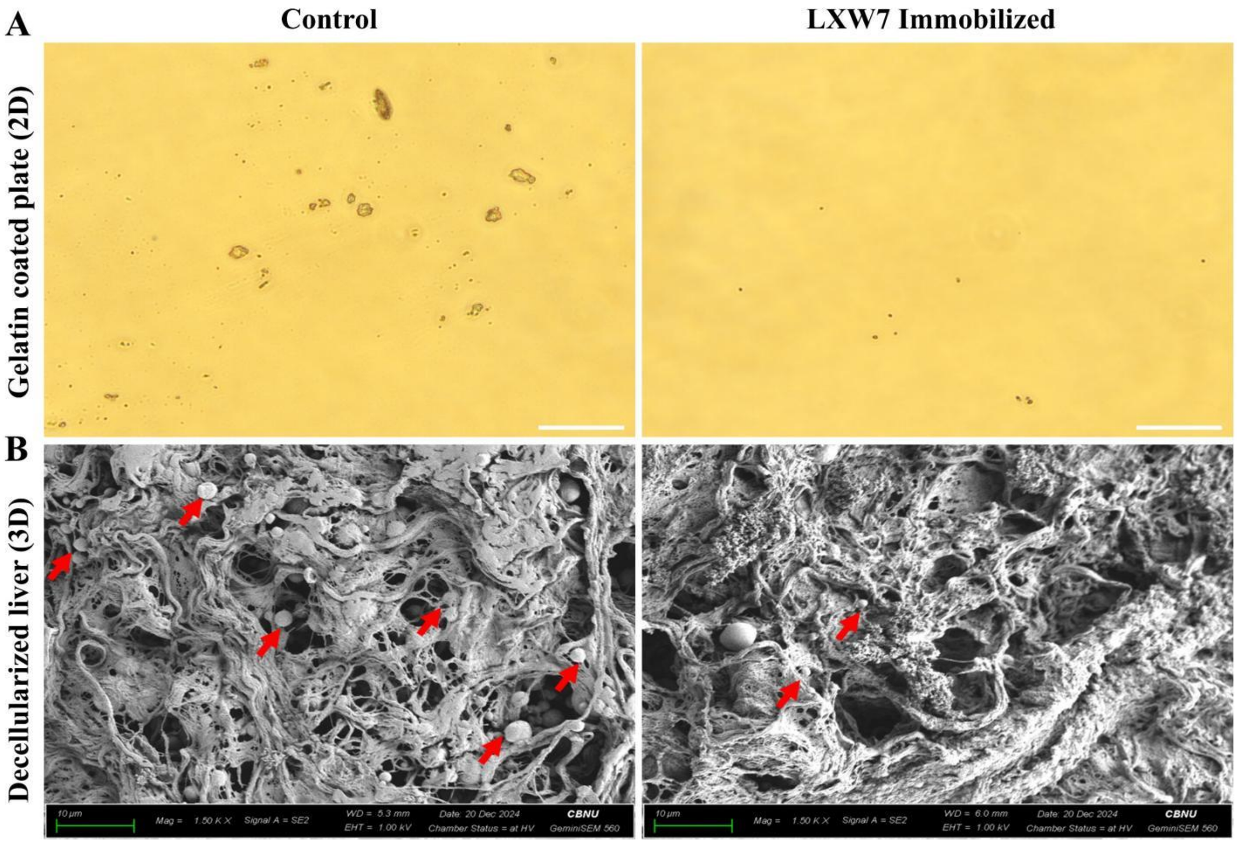

Figure S3. Platelet adhesion assay on gelatin-coated plates and DLS scaffolds. (**A**) Representative images showing platelet attachment on gelatin-coated surfaces and (**B**) DLS scaffolds following incubation with PRP. Compared with controls, LXW7-coated surfaces exhibited markedly reduced platelet adhesion, indicating the anti-platelet properties of LXW7 functionalization. Scale bars= 500 µm.

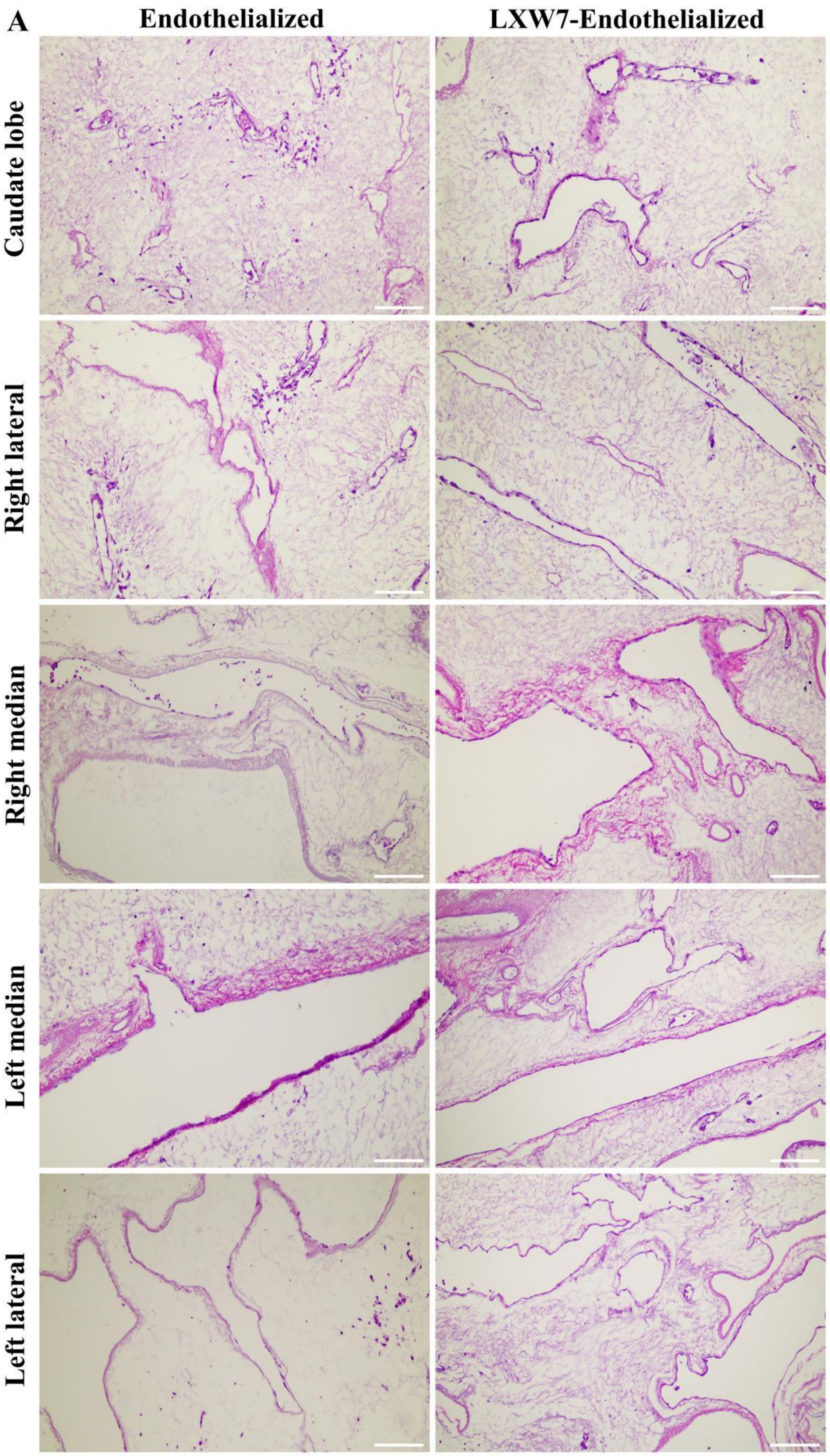
A
Endothelialized
LXW7-Endothelialized
Caudate lobe
Right lateral
Right median
Left median
Left lateral

Figure S4. An overview of the Endothelialized vessels. (**A**) H and E staining images of different lobes of the endothelialized scaffolds. LXW7 endothelialized scaffold showing well-spread monolayers of HUVECs adhered to the vessels, while incomplete vessels lined the case of the endothelialized group. Black arrows indicate cell adherence to the blood vessels. Scale bar=200μm.

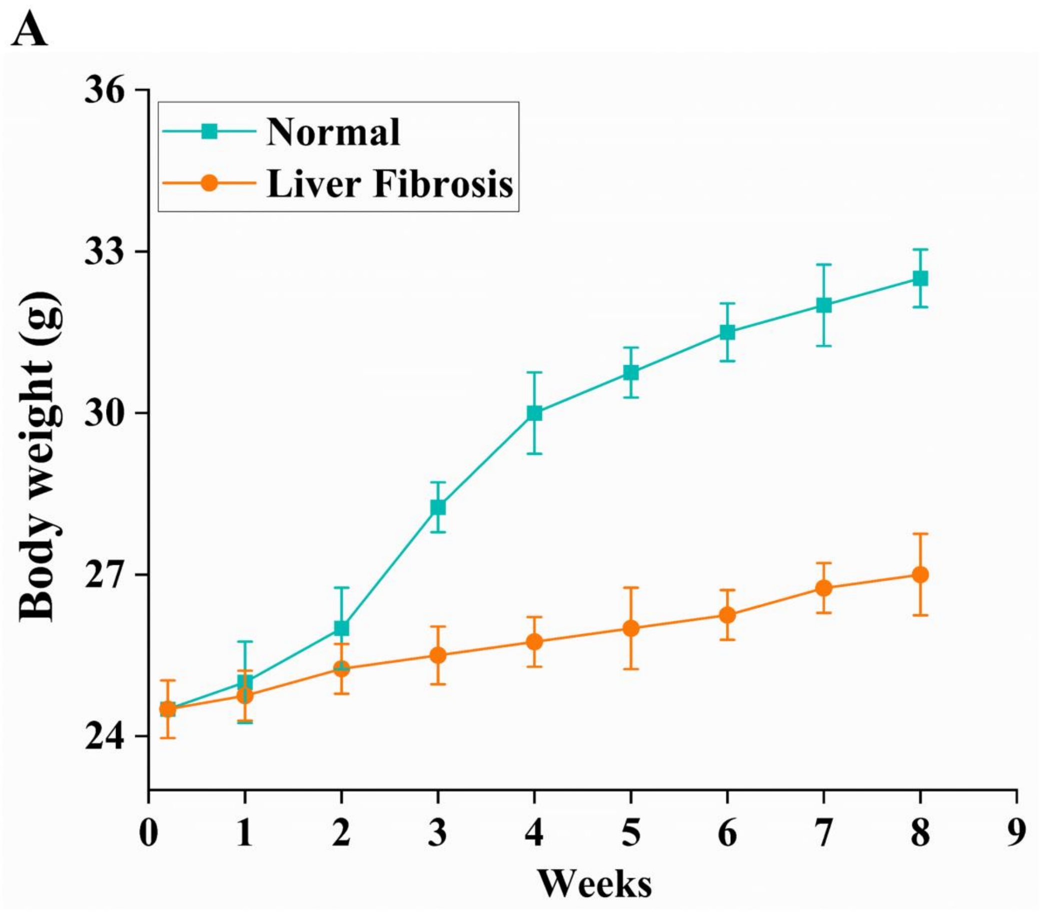

Figure S5. Body weight of normal and liver fibrotic mice. (**A**) Body weight monitoring showed impaired growth in TAA-treated mice compared to controls (n = 4 mice per group).
